# Anxiety levels among health sciences students during their first visit to the dissection room

**DOI:** 10.1186/s12909-020-02027-2

**Published:** 2020-04-09

**Authors:** Carmen Romo-Barrientos, Juan José Criado-Álvarez, Jaime González-González, Isabel Ubeda-Bañon, Alicia Flores-Cuadrado, Daniel Saiz-Sánchez, Antonio Viñuela, Jose Luis Martin-Conty, Teresa Simón, Alino Martinez-Marcos, Alicia Mohedano-Moriano

**Affiliations:** 1 Integrated Care Management at Talavera de la Reina, Castilla-La Mancha Health Services, Talavera de la Reina, Toledo, Spain; 2 Department of Medical Sciences, School of Health Sciences, University of Castilla-La Mancha, Avenida Real Fábrica de las Sedas, s/n 45600 Talavera de la Reina, Toledo, Spain; 3 Department of Medical Sciences, School of Medicine, Regional Centre for Biomedical Research, University of Castilla-La Mancha, Ciudad Real, Spain; 4 Department of Nursing, Physiotherapy and Occupational Therapy, School of Health Sciences, University of Castilla-La Mancha, Talavera de la Reina, Toledo, Spain; 5 Department of Psychology, School of Health Sciences, University of Castilla-La Mancha, Talavera de la Reina, Toledo, Spain

**Keywords:** Anatomy education, Dissection, Prosection, Anxiety, Health sciences

## Abstract

**Background:**

The teaching of human anatomy is often based on practices of cadaver dissection and prosected specimens. However, exposure to human cadavers might be stressful and anxiety-inducing for students. The aim of this study is to explore the degree of satisfaction and anxiety among first-year students in the Medicine, Occupational Therapy, Speech Therapy and Nursing programmes at the Universidad de Castilla-La Mancha (Spain) who are experiencing their first dissection/prosection practice to develop stress coping strategies.

**Methods:**

A total of 204 health sciences students participated in this study. The State-Trait Anxiety Inventory was used to evaluate anxiety.

**Results:**

‘State Anxiety’ (SA) decreased significantly throughout the course (*p* < 0.05), from 20.7 ± 19.29 to 13.7 ± 11.65 points. Statistical differences (p < 0.05) in SA were found between the different health sciences, and pre-practice SA was significantly different from post-practice SA. The students with the highest pre-practice SA levels were nursing students (31.8 ± 33.7 points), but medical students had the highest post-practice SA levels (18.4 ± 12.82 points).

**Conclusions:**

Although students were satisfied with dissection practices (96.8% of them recommended that the practices be retained for future courses), the experience can provoke stressful responses that must be addressed using advanced preparation and coping mechanisms, especially among medical and nursing students.

## Background

Human anatomy is an essential component of the educational curriculum in many health sciences university programmes [1–6]. Dissection or prosection practices are usually associated with the teaching of anatomy. The use of cadavers in these practices can be a stressful experience. Indeed, 30% of students believe that the dissecting room is a stressful environment, with 4% of students suffering post-traumatic stress. However, 76% of students agree that this experience is irreplaceable [7, 8]. Medical students prefer practical anatomy sessions (via dissection and prosection) over theoretical classes (via didactic teaching or models) [9]. In the last decade, anatomy teaching in the dissecting room has changed considerably in terms of technology, infrastructure and safety. However, we have not considered how conducting these practices routinely in universities affects anxiety levels among students, specifically at the University of Castilla-La Mancha (UCLM). The reactions and feelings witnessed in dissecting rooms have been studied in different countries and for different health sciences fields [10–19], and researchers have examined anxiety associated with the dissection of the human body [20]. We also need to assess the kinds of reactions and feelings that dissection/prosection could cause before adding this activity to the new curricular design of the Human Anatomy courses in our health sciences programmes, especially in those programmes where these practices have not been standard, e.g., nursing, speech therapy, and occupational therapy. This preliminary research would help in the development of strategies to improve our students’ academic and clinical skills [21] and would have a positive impact on their learning. Some studies have shown that using coping strategies can help reduce the anxiety caused by these practices [13, 22–25]. This study explores the levels of anxiety experienced by first-year medicine, occupational therapy, speech therapy and nursing students at UCLM in relation to their first practical dissection/prosection class. This preliminary information will help us design specific strategies to reduce students’ stress and improve their academic results.

## Methods

This descriptive, cross-sectional study was conducted with first-year medicine (MED), occupational therapy (OT), speech therapy (ST) and nursing (NUR) students who enrolled in human anatomy courses at the UCLM Medical School in Ciudad Real (Spain) and the UCLM School of Health Sciences in Talavera de la Reina (Toledo, Spain) during the 2016–2017 academic year. The MED students had never previously participated in a practical dissection class with cadavers, and the ST, OT, and NUR students had not participated in a prosection class. The main objective of prosection and dissection was to study anatomical structures and the relationship between them. In particular, NUR students studied the “apparatus and systems, especially the circulatory, nervous and urinary systems”, OT students studied the “locomotor and nervous systems”, ST students studied the “head and neck, respiratory and nervous systems”, and the MED students studied all apparatus and systems.

Participation in the classes was compulsory for MED students and voluntary for ST, OT and NUR students. Before the practice, the students were informed about the general aims of the study, and their anonymity was guaranteed after completing the consent forms. Ethical approval for this study was granted by the Ethical Committee for Clinical Research of Talavera de la Reina (Toledo, Spain) (Code: 23/201/). Prior to entering the dissection room, all students received their corresponding individual protection kits. The students were informed about the general health and safety norms regarding the procedures for cadaver donation.

Two anonymous questionnaires (“ad hoc”, STAI) were carried out before and after the first practice. The “ad hoc” questionnaire assessed each student’s feelings, emotions and satisfaction with this practice [15, 17]. These questionnaires were completed by the students themselves so that they could be combined with a second questionnaire that was completed immediately after the practice.

The State-Trait Anxiety Inventory (STAI, adapted to Spanish) [19] was used to evaluate ‘State Anxiety’. The STAI is a self-reported instrument conceived to assess anxiety in healthy adults. It contains two scales that measure two distinct, but related, anxiety types: ‘State Anxiety’ (SA) and ‘Trait Anxiety’ (TA). TA measures an individual’s usual or base emotional state. SA, on the other hand, evaluates subjective, variable and transitory feelings of tension, apprehension and fear, thus assessing how a person feels in a given situation (e.g., before a practical dissection class). Each scale contains 20 questions, providing a numerical score for each anxiety type. The results are converted into a numerical scale from 0 to 10 according to gender and age (19 years old or over). An SA score higher than 6 (SA > 6) indicated that the practical dissection class caused anxiety in students [10, 19, 26]. The STAI has been validated for use with a Spanish population and has a Cronbach’s alpha of 0.93 and 0.92 for TA and SA, respectively [27].

The SA questionnaire was administered to students before and immediately after their first dissection (MED students) or prosection practice (NUR, OT, and ST students); the TA was only completed before the practice.

Additionally, several questions about the degree of satisfaction with the class and its quality were also included [15, 17, 18].

A 5% confidence level was established. The statistical software SPSS Version 15.0 for Windows was used to analyse the data (SPSS Inc. Released 2006. SPSS for Windows, Version 15.0. Chicago, SPSS Inc.).

## Results

A total of 204 (100%) students answered the initial questionnaire, including 42 NUR students (20.6%), 47 ST students (23%), 57 MED students (27.9%) and 58 OT students (28.4%). Sixteen (7.8%) students who did not complete the final questionnaire were excluded from the study (no differences across programmes). The mean age for students was 19 ± 2.43 years (median: 19 years), and 164 (87.7%) were female; the distributions were similar across different programmes.

SA decreased significantly (*p* < 0.05) after the practice, from 20.7 ± 19.29 to 13.7 ± 11.65 points. Male students started with higher SA levels (21.5 ± 26.49 points) than female students (20.1 ± 17.92 points), but after the practice, females had higher SA levels (11.2 ± 11.31 for males and 13.6 ± 11.53 for females); these decreases of 10.4 and 6.83 points, respectively, were not statistically significant (*p* > 0.05).

**Fig. 1:**
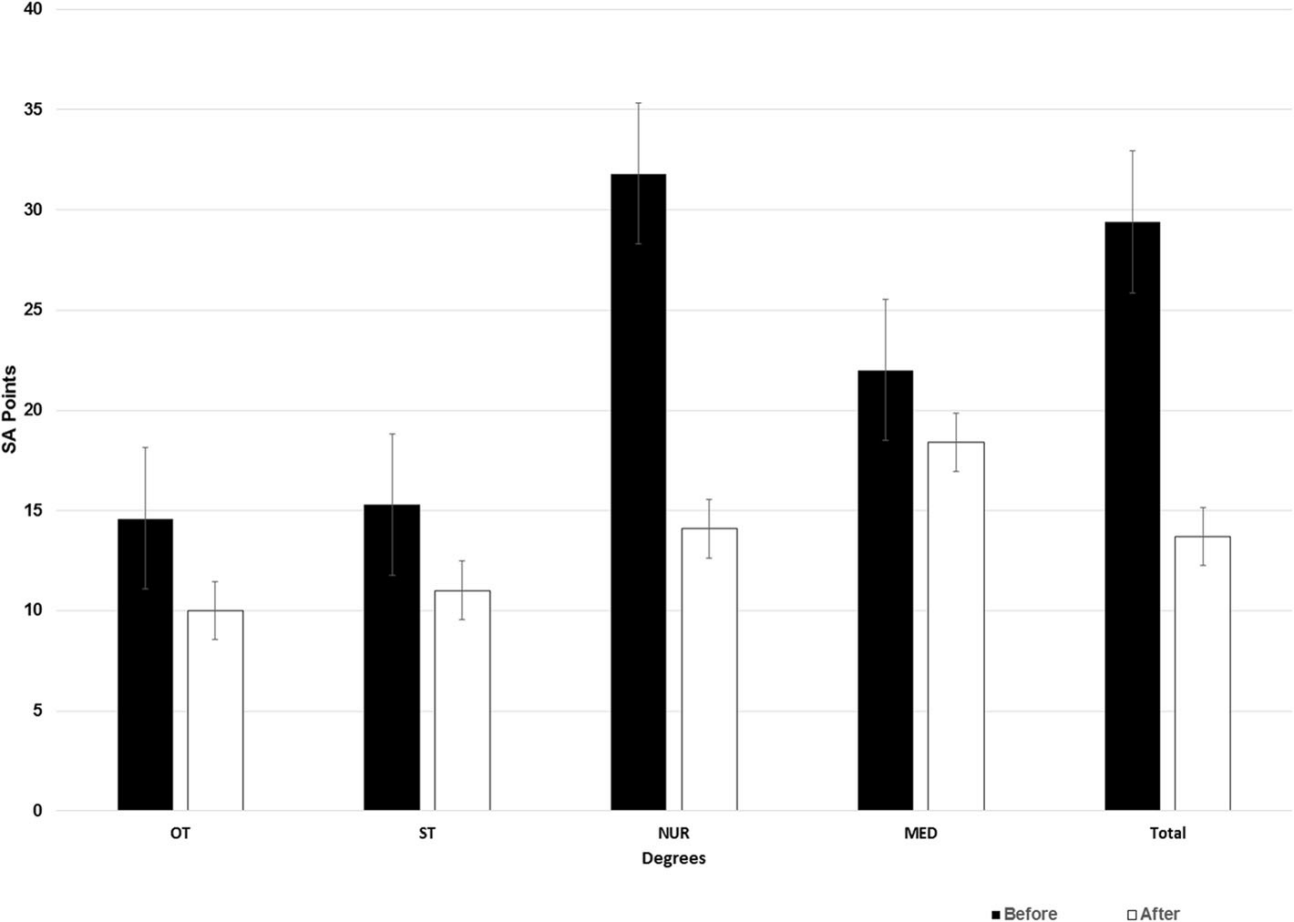
Percentage of ‘State Anxiety’ (SA) by degree and total

Statistically significant differences (p < 0.05) in both pre-practice and post-practice SA levels were observed between the different programmes (Fig. 1). NUR students had the highest SA levels before the practice, at 31.8 ± 33.7 points, and MED students had the highest SA levels after the practice, at 18.4 ± 12.82 points (Fig. 1). Students’ mean TA at the start of the course was 22.2 ± 17.29 points; no statistically significant difference was observed between genders (*p* > 0.05), with a pre-practice TA of 24.7 ± 25.22 and 21.9 ± 16.51 points in males and females, respectively, and a post-practice TA of 14.3 ± 9.99 and 18.6 ± 10.98 points in males and females, respectively (*p* > 0.05). The percentage of students who reached a cutoff score of 6 on the SA scale and thus had anxiety increased from 19.9% (N: 39) before the practice to 22.3% (N: 42) after the practice, and the SA scores were significantly different across programmes (*p* < 0.05) but not across genders (*p* > 0.05).

A statistically significant correlation was observed between the SA and TA levels before the first practice, with a Pearson’s correlation coefficient of 0.716 (*p* < 0.05); the correlation after the practice was 0.763 (*p* < 0.05). The correlation between pre-practice SA and post-practice SA was 0.218 (*p* < 0.05). A total of 96.8% of the students (N: 180) recommended that the practices be retained for future courses; there were no statistically significant differences across genders (*p* > 0.05) or programmes (*p* > 0.05). In general terms, 98.3% (N: 200) of students were “Satisfied” or “Very satisfied”, and 85.1% (N: 174) were curious about the practices. The mean overall satisfaction level was 8.8 ± 1.18 points (median: 9) on a 1–10 scale.

## Discussion

For many medical students, exposure to human bodies in the anatomy lab is their first exposure to death. Practical classes using cadavers can give rise to a series of uncomfortable and stressful experiences for health science students [8, 10, 11, 14, 15, 24, 28]. One-fifth of the students felt anxiety (SA) before they entered the dissecting room at the beginning of the course, and this figure rose slightly after the practice. These data are consistent with previous findings of 14.4% [22] and 17.8% [29]. This situation is the same for all our students, especially NUR students (31.8 points). Students’ mean SA decreased significantly (*p* < 0.05) after the session, from 20.7 to 13.7. These data were consistent with the findings of Casado [30], who reported a decrease from 24.7 points to 16.7 points, and the findings of Arraez [10], who reported a decrease from 26.6 points to 14.2 points.

On the other hand, SA was higher in female students after the practice, whereas for male students, it was the opposite. This has been reported by other authors: baseline anxiety levels, measured as TA, are similar for both genders, but the practical dissection class causes anxiety, and this anxiety is slightly higher in females [14, 22, 28]. Other studies that do not use the STAI have reported differences in anxiety between genders [14, 21, 31, 32]. Wisenden [33] indicated that female students experienced greater anxiety than male students when exposed to cadavers. As a consequence, women adapt less quickly to the new situation of the dissection course than men and more frequently request an introductory course [13, 34].

The decrease in SA among our students cannot be due to what some authors have considered to be a coping phenomenon and the avoidance of adverse reactions, as there was no time for such phenomena between the questionnaires; rather, the students had to deal with their preconceived ideas about the practice they were about to face [10, 12, 22, 25, 28, 35]. Arraéz-Aybar showed that anxiety levels tend to decrease as medical students perform more practices and dissections, reducing from 26.62 to 14.34 with baseline values of 14.3 to 13.1 [10, 22], consistent with our results. Some studies have not indicated any changes in SA levels, with scores of approximately 30 points throughout the course; this finding was attributed to the students’ interest in completing the course [36]. Students’ anxiety before their first dissection practice is determined by the student’s situation and is measured as TA. Later, the individual and personal differences, measured as SA, allow student reactions to be predicted [26]. The figures for both SA and TA are similar to those published in a study conducted on occupational therapists, with mean figures of 24.1–26.1 points before the start of the course and 12.2–21.7 points afterwards [26]. These figures differ considerably from the 42.6 points for TA and the 46.7 for SA obtained in a study conducted on medicine, dental and pharmacy students [11]. When faced with a stressful situation such as a practical Human Anatomy lesson, TA and SA are strongly correlated prior to the practice (0.716). Afterwards, however, the correlation drops to 0.207, and anxiety is found only in those students who presented higher levels before the practice. Several studies have found differences in anxiety levels between different programmes, and although they do not suggest any reasons to explain these differences, as in our study, they find that MED students have higher mean SA scores (18.1 ± 12.82) after the practice, while OT students (10 ± 6.71) have the lowest scores [18]. A possible explanation for this pattern could be that MED students assume more responsibility and are more aware of the importance of these practical lessons for their future professional practice [11, 22, 25, 28, 31]. However, ST and OT students only see these practices as a means for learning about anatomical structures. In addition, NUR students have greater training in empathy and emotional intelligence, with their roles being associated with compassion and care [37–40], which could explain their greater empathy towards the cadaver and their greater anxiety both before and after the practice. MED and NUR students may have given greater thought to death and its meaning due to their roles, and the acceptance of these and derived issues, such as concerns about the brevity of life and uncertainty about what happens after death, may increase their anxiety [39, 41–43]. Therefore, the detection of anxiety should also be done beforehand [13, 22–24], especially with MED and NUR students [41]. For these students, it may be necessary to incorporate coping techniques in their dissection and prosection practices [5], such as the use of audio-visual aids to explain dissection rules, procedures and familiarization with cadaver donation [10], the use of background music in the dissecting lab [44], or humanizing the students’ encounter with a human cadaver by reflecting about life and death [45]; additionally, understanding the personalities of our students can help those who are anxious about the dissecting room experience [21, 46]. These methods could be extrapolated to other educational environments where students report having feelings of fear and anxiety, such as the surgery room or the autopsy room [47–50].

On the other hand, there is no evidence in the literature suggesting that dissection practices generate more anxiety than prosection practices; however, there is scientific evidence suggesting that there are no significant differences in student evaluations of dissection and prosection of cadavers [51, 52]. The use of dissection practices is more frequent for MED, and prosection practices are used more in other health sciences programmes. Nnodim [52] showed that prosection was more profitable and required less time to learn the same amount of material. The content and temporality of the anatomy in NUR, TO and ST programmes at our university is much lower than that in MED programmes, which is why NUR, TO and ST programmes choose prosection.

Despite any drawbacks, the students clearly valued this experience highly and showed great curiosity about this activity, with values similar to those published by Arraéz-Aybar (88, 5%) [22, 28]. The experience appeared to effectively support their personal progression as learners with respect to the professional and clinical applications of this knowledge [4, 8, 41], improved their skills and attitudes with regard to their future professions [53], helped them confront death [42], and promoted leadership and teamwork [54–57]. In addition, dissection offers students a unique opportunity to explore the human body in a hands-on manner while also putting into practice the theoretical aspects of their education. Overall, our students indicated that they were satisfied or very satisfied with the experience and would recommend it for future courses, consistent with findings from other studies [14, 41].

### Limitations of the study

The limitations to this study were that no previous results were available with which to compare these findings, since this was the first year that students’ anxiety levels were evaluated and the first time they worked with a cadaver.

On the other hand, it should be noted that for MED students, these practices were compulsory and took place throughout the course (100–120 h per year), while for other health sciences students (OT, ST, NUR), the practices were occasional (10–15 h per year) and voluntary. In addition, MED students carry out dissections, whereas OT, ST and NUR students use prosected specimens. The findings can be generalized to other Spanish universities, although it is difficult to know if the findings would hold in other countries due to cultural, religious, philosophical and educational differences.

## Conclusions

Dissection practices provide many benefits in the teaching of anatomy. Overall, our students positively value this activity and would recommend it for future courses. It should be taken into account that the experience of entering a dissecting room can challenge some students emotionally. It is important that students with higher anxiety levels are armed with coping techniques to help reduce stress, especially with MED and NUR students. Therefore, it is necessary to measure anxiety at the beginning of the anatomy course. Traditionally, MED and NUR students will have formed greater empathy towards patients, which can explain their greater anxiety in the presence of a cadaver. Additionally, for OT and ST students, prosection may be of minor relevance to their future professional life; however, for MED and NUR students, it is a more meaningful occasion that makes them face up to the realities of their future professional practice.

The possibility of experiencing the death of a human being in a controlled environment is a learning opportunity for health science students.

## Data Availability

The datasets used and/or analysed during the current study available from the corresponding author on reasonable request.

## Abbreviations

MED: Medicine
NUR: Nursing
OT: Occupational Therapy
SA: State Anxiety
ST: Speech Therapy
STAI: State-Trait Anxiety Inventory
TA: Trait Anxiety
UCLM: University of Castilla-La Mancha
